# 2-Aminopurine Enhances the Oncolytic Activity of an E1b-Deleted Adenovirus in Hepatocellular Carcinoma Cells

**DOI:** 10.1371/journal.pone.0065222

**Published:** 2013-06-04

**Authors:** David Sharon, Michael Schümann, Sheena MacLeod, Robyn McPherson, Shyambabu Chaurasiya, Andrew Shaw, Mary M. Hitt

**Affiliations:** 1 Department of Oncology, University of Alberta, Edmonton, Alberta, Canada; 2 Institut für Virologie, Klinikum der Philipps-Universität Marburg, Marburg, Germany; University of Birmingham, United Kingdom

## Abstract

Adenoviruses with deletions of viral genes have been extensively studied as potential cancer therapeutics. Although a high degree of cancer selectivity has been demonstrated with these conditionally replicating adenoviruses, low levels of virus replication can be detected in normal cells. Furthermore, these mutations were also found to reduce the activity of the replicating viruses in certain cancer cells. Recent studies have shown that co-administration of chemotherapeutic drugs may increase the activity of these viruses without affecting their specificity. We constructed an adenovirus with deletions of both the E1b and the VA-RNA genes and found that replication of this virus was selective for human hepatocellular carcinoma (HCC) cell lines when compared to normal cell lines. Furthermore, we show that 2-aminopurine (2′AP) treatment selectively enhanced virus replication and virus-mediated death of HCC cells. 2′AP did not compensate for the loss of VA-RNA activities, but rather the loss of an E1b-55K activity, such as the DNA damage response, suggesting that co-administration of 2′AP derivatives that block host DNA damage response, may increase the oncolytic activity of AdΔE1bΔVA without reducing its selectivity for HCC cells.

## Introduction

Adenoviruses with deletions or mutations within the early region 1b (E1b) gene have been shown to replicate selectively in cancer cells. The most commonly studied cancer selective oncolytic adenovirus is Ad-dl1520 (Onyx-015) [1]. Due to the role of the E1b-55K protein in the inhibition of p53 [2]–[4], selectivity was first thought to be due primarily to inactivating mutations or deletions of the p53 gene in cancer cells, thus relieving the requirement for E1b-55K in virus replication [5]–[8]. However, the cancer selectivity was later found to be independent of p53 and it is currently thought that loss of other functions of E1b-55K may confer viral selectivity to cancer cells [9]–[12]. One of these functions is to inhibit the DNA damage response. Sensing the linear viral DNA genome as double-stranded (ds) DNA breaks activates the DNA damage response pathway, which in turn, activates checkpoint proteins that block further DNA replication of both host and viral DNA [13], [14]. Furthermore, in an attempt to repair the damage, host proteins can induce concatemerization of viral genomes, which produces DNA sequences larger than the packaging limit [14]–[16]. Several viral proteins have been shown to block activation of the DNA damage response, such as E1a, E4orf3 and E1b-55K in association with E4orf6 [17]–[21]. In particular, two cysteine residues of E1b-55K (C454 and C456) are important in the inhibition of the DNA damage response through inhibition of at least 2 key proteins within the pathway, Mre11 and DNA ligase IV [17], [18].

In addition to E1b-55K deletion, adenoviruses with deletions of the E1b-19K gene were also shown to be oncolytic [22], [23]. Similar to E1b-55K, E1b-19K has a role in the inhibition of premature virus-mediated cell death, therefore, E1b-19K deletion is thought to increase virus-mediated killing. Furthermore, adenoviruses with deletions of both E1b-19K and E1b-55K were found to have increased selectivity for cancer cells when compared to adenoviruses with a single deletion of either E1b-19K or E1b-55K [24], [25].

In addition to the E1b deletions, deletions of other adenoviral genes were shown to allow selective virus production in cancer cells, such as the deletion of the virus-associated RNA (VA-RNA) genes [26]–[28]. These genes express two non-coding RNA molecules (VA1 and VA2). Although the role of VA2 in virus replication is largely unknown, VA1 is thought to be important for inhibiting the activation of the interferon response, an important cellular antiviral response [29], [30]. This inhibition occurs through direct binding and inactivation of RNA sensors that activate the interferon response, such as PKR [31]–[33]. Activated PKR can inhibit both viral and cellular protein synthesis through phosphorylation of eIF2α, as well as induce premature cell death during virus infection [34]–[36]. Activating ras mutations, which are found in many cancer cells, block PKR phosphorylation of eIF2α. Therefore, cancer cells with activating ras mutations have been hypothesized to support VA-RNA deleted adenovirus replication [26], [37].

The adenine analog 2-aminopurine (2′AP) alters a number of pathways that are important in adenoviral infection. It was shown to block PKR activity, thus blocking shutdown of protein synthesis [38]. 2′AP was also shown to inhibit interferon-stimulated gene expression, which is a downstream effect of the interferon response [39], [40]. Several studies have shown that 2′AP can also inhibit ATM and ATR, proteins within the DNA damage response, which are activated by Mre11 [41]–[43]. Furthermore, the expression and activity of p53 following DNA damage were found to decrease in cells treated with 2′AP. Interestingly, PKR directly interacts with and activates p53 following DNA damage [44], [45], and p53 induces PKR expression [46], suggesting a possible convergence between the interferon response and the DNA damage response. Recent reports showed that, in addition to the VA-RNAs, E1b-55K can also inhibit PKR and eIF2α phosphorylation as well as inhibit the activation of interferon-stimulated genes following adenoviral infection [47], [48]. Due to the ability of 2′AP to rescue the replication of a VA-RNA-deleted adenovirus in certain cells, the drug may also be able to rescue the replication of adenoviruses deleted of both VA-RNA and E1b-55K sequences.

We report here the construction and assessment of E1b-deleted adenoviruses with or without additional deletion of the VA-RNA genes (Ad**Δ**E1b**Δ**VA and Ad**Δ**E1b, respectively). A previous study has shown that increased E1a expression levels in cancer cell lines can increase E1b-deleted virus replication [49]. Therefore, the E1a gene in our construct was placed under the control of the strong immediate early murine cytomegalovirus (mCMV) promoter [50].

Through growth analysis of Ad**Δ**E1b and Ad**Δ**E1b**Δ**VA in HCC and normal cell lines we found that, while the deletion of VA-RNA attenuated virus replication in normal cells, VA-RNAs were dispensable for E1b-deleted virus replication in the HCC cell lines, Hep3B and HepG2. Due to the effect of 2′AP on both the interferon response and the DNA damage response, we examined whether this drug could rescue the replication of E1b-deleted adenoviruses either encoding or not encoding VA-RNAs. Surprisingly, although 2′AP treatment increased the replication of both Ad**Δ**E1b and Ad**Δ**E1b**Δ**VA in normal MRC5 cells, the drug was unable to rescue Ad**Δ**E1b**Δ**VA replication to the same level as Ad**Δ**E1b in these cells. In contrast, 2′AP treatment increased replication of both Ad**Δ**E1b and Ad**Δ**E1b**Δ**VA to similar high levels in HepG2 cells. Furthermore, we found that the E1b-55K domain required for the inhibition of host DNA damage response was important for adenovirus replication in HepG2 cells, and that 2′AP compensates for the loss of this domain.

## Materials and Methods

### Cell Lines

Hep3B (provided by Dr. Roseline Godbout, University of Alberta) and HepG2 (ATCC HB-8065) hepatocellular carcinoma cell lines, as well as WI-38 lung fibroblasts (ATCC CCL-75), were grown in DMEM high glucose (Gibco). MRC5 lung fibroblasts (ATCC CCL-171) and HEK293 E1-transformed human embryonic kidney cells [51] (provided by Dr. Frank Graham, McMaster University) were grown in MEM (Gibco). All media were supplemented with 10% FBS (Gibco), 2 mM L-glutamine (Gibco) and 1× antibiotic-antimycotic (100 units of penicillin, 100 µg streptomycin, 0.0085% fungizone) (Gibco).

### Viral Constructs

**Figure 1:**
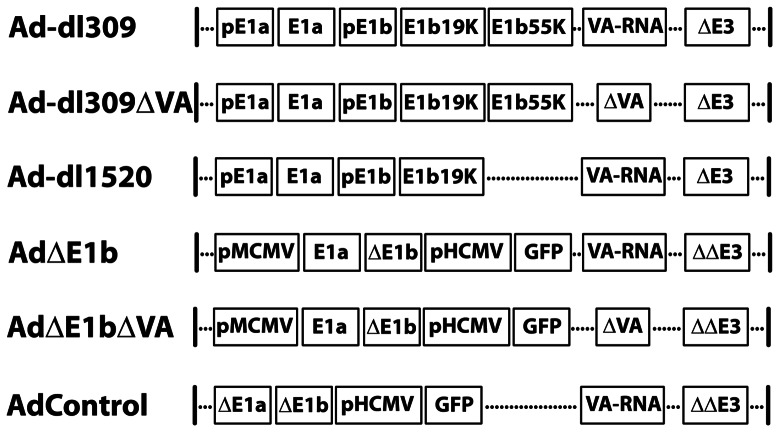
Schematic representation of the adenoviral genomes used in this study. Ad-dl309 encodes both E1a and E1b genes including their respective Ad promoters (pE1a and pE1b, respectively). The virus has a deletion in the E3 region spanning 30005–30750 bp [81]. Ad-dl309**Δ**VA and Ad-dl1520 have additional deletions in the VA-RNA genes (10667–10702 bp as well as 10929–10943 bp) or in the E1b-55K gene, respectively. Ad**Δ**E1b, Ad**Δ**E1b**Δ**VA and AdControl (**Δ**E1a**Δ**E1b) were constructed using the AdEasy-1 system and the pAdTrack shuttle plasmid. The pAd-Easy-1 plasmid has a larger deletion in the E3 region spanning 28130–30820 bp. The pAd-Track plasmid has E1 sequences spanning 480–3533 bp replaced with the EGFP gene under the control of the human cytomegalovirus immediate early (hCMV) promoter [53]. The E1a gene under the control of the murine cytomegalovirus immediate early (mCMV) promoter was introduced back into the genomes of Ad**Δ**E1b and Ad**Δ**E1b**Δ**VA. Additionally, Ad**Δ**E1b**Δ**VA has the same VA-RNA deletions as Ad-dl309**Δ**VA.

Ad-dl309, Ad-dl309**Δ**VA (dVAs) and the non-replicating AdControl (rAd-gal-GFP) [27], [52] were kind gifts from Dr. Matthias Dobbelstein (Göttingen University). Ad-dl1520 [1] was a kind gift from Dr. Philip Branton (McGill University). Ad**Δ**E1b and Ad**Δ**E1b**Δ**VA were constructed using the AdEasy-1 system [53]. Briefly, Ad5 E1a gene (excluding viral E1a promoter) was PCR amplified from the plasmid pXC1 (nucleotides 542–1564; a gift from Dr. Frank Graham). This fragment was added to an expression vector encoding a PacI-deleted murine cytomegalovirus immediate early (mCMV) promoter. The expression cassette was then transferred into the pAd-Track shuttle vector. This pAd-Track plasmid was then recombined in BJ5183 bacterial cells with the pAd-Easy-1 plasmid or a derivative lacking the VA-RNA genes [27]. The resulting recombinant plasmids were cleaved with PacI to release the viral genome and used to transfect HEK293. Recombinant viruses were isolated and amplified in Hep3B cells due to the reduced growth of VA-RNA deleted viruses in HEK293 cells. Ad-dl309 and Ad-dl309**Δ**VA were also amplified in Hep3B cells, while the replication-deficient AdControl was amplified in HEK293 cells. All viruses (Figure 1) were purified by CsCl gradient sedimentation [54] and their genomes were isolated and verified by restriction enzyme digestion. The concentration of virus particles was determined spectrophotometrically [54]. The concentration of infectious virus was determined through plaque assays performed on HEK293 cells as previously described [54]. Due to the inability of Ad**Δ**E1b**Δ**VA to produce plaques in HEK293 cells, the concentration of infectious units of Ad**Δ**E1b**Δ**VA as well as Ad**Δ**E1b were determined from the number of GFP-positive Hep3B cells observed 3 days after infection with limiting dilutions of virus (designated green fluorescent units, GFU). The ratios of virus particle to infectious units of Ad**Δ**E1b and Ad**Δ**E1b**Δ**VA were approximately 70, similar to the particle to PFU ratio for the other viruses.

### Virus Replication Assays

Confluent cells in a 24 well plate were infected with Ad-dl309, Ad-dl309**Δ**VA, Ad-dl1520 or Ad**Δ**E1b at MOI of 1 PFU/cell (or 1 GFU/cell where indicated) in 0.2 mL PBS in duplicate. One hour post-infection fresh medium was added either with or without 2.5 mM 2′AP (Invivogen, San Diego, CA, USA). Infected cells and media were harvested at the indicated times, and subjected to 3 rounds of freeze-thaw cycles. For the virus release assay, the media from infected wells were also harvested and centrifuged at 1000 rpm for 5 minutes prior to 3 freeze-thaw cycles. Virus concentrations were determined by plaque assay (in duplicate) in HEK293 cells, or where indicated, by limiting dilution assay on Hep3B cells (in triplicate) as described above.

### E1b Complementation Assay

In order to determine the effect of the E1b-55K expression vector on viral replication, HepG2 cells were stably transfected with wild-type pE1b-55K or pE1b-55K-Mut (C454S/C456S) plasmid DNA [17] (a kind gift from Dr. Thomas Dobner, Heinrich-Pette Institute) using selection medium containing G418 at the concentration of 1 µg/mL. The two E1b-55K expressing cell lines (HepG2-E1b-WT and HepG2-E1b-Mut) as well as parental HepG2 cells were infected with Ad-dl309, Ad-dl1520 or Ad**Δ**E1b. One hour post-infection fresh medium was added either with or without 2.5 mM 2′AP. Infected cells and media were harvested at the indicated times and subjected to 3 freeze-thaw cycles. Infectious units were determined as either PFUs or GFUs as indicated.

### Cell Survival Assay

Cells were transferred into 96 well plates at 5000 cells/well. The following day, the medium was removed and cells were infected at the indicated concentrations in quadruplicate. One hour later, fresh medium was added with or without supplementation of 2.5 mM 2′AP. Six days post-infection, Alamar Blue (Resazurin; Sigma, St Louis, MO, USA) was added to a final concentration of 44 µM. Fluorescence of each well was measured (excitation at 544 nm; emission at 590 nm) using the FLUOstar Omega plate reader (BMG Labtech, Ortenberg, Germany).

### Western Blot Analysis

Cells were infected at MOI of 100 virus particles per cell (VP/cell). Two and four days post-infection, cells were harvested and lysed with RIPA buffer supplemented with 1 mM PMSF (Sigma) and 1x Protease Inhibitor Cocktail (Sigma). 10 µg protein samples were electrophoresed on an SDS polyacrylamide gel then transferred to a nitrocellulose membrane (Biorad, Mississauga, ON, CA). Membranes were blocked with 5% powdered skim milk in PBST (PBS with 0.5% Tween-20) for 1 hour and incubated overnight at 4°C with antibodies against E1a (M73, NeoMarkers, Fremont, CA, USA), fiber (RDI-Adenov2Abm, Research Diagnostics Inc, Flanders, NJ, USA), β-actin (MA5–15739, Thermo Fisher Scientific Inc, Rockford, IL, USA) or p53 (Pab 1801, Santa Cruz Biotechnology, Santa Cruz, CA, USA). Membranes were washed in PBST and incubated for 1 hour in horseradish peroxidase conjugated secondary antibodies (Jackson ImmunoResearch Laboratories, West Grove, PA, USA).

### p53-activated Luciferase Reporter Assay

HepG2 and Hep3B cells in a 24 well plate were transfected with 1 µg of p53-reporter expression vector (Panomics, Fremont, CA, USA) using Lipofectamine 2000. The following day, cells were infected with Ad-dl309, Ad-dl1520 or Ad**Δ**E1b at MOI of 100 VP/cell for 1 hour in PBS, followed by addition of fresh medium or medium containing 2.5 mM 2′AP. Two days post-infection, cells were washed with PBS and lysed, and firefly luciferase levels were measured using the Dual Luciferase Reporter Assay System (Promega) with the FLUOstar Omega plate reader (BMG).

### GFP Fluorescence Intensity Measurements

Cells were transferred into 96 well plates at a concentration of 5000 cells per well. The following day, the medium was removed and cells were infected at MOI of 100 VP/cell in quadruplicate. One hour later, fresh medium or medium with 2.5 mM 2′AP was added to the infected cells. GFP fluorescence was determined with the FLUOstar omega plate reader (BMG) at the indicated time-points post-infection.

## Results

### Replication Properties of VA-RNA- or E1b-55K-deleted Adenoviruses in HCC and Normal Cell Lines

**Figure 2:**
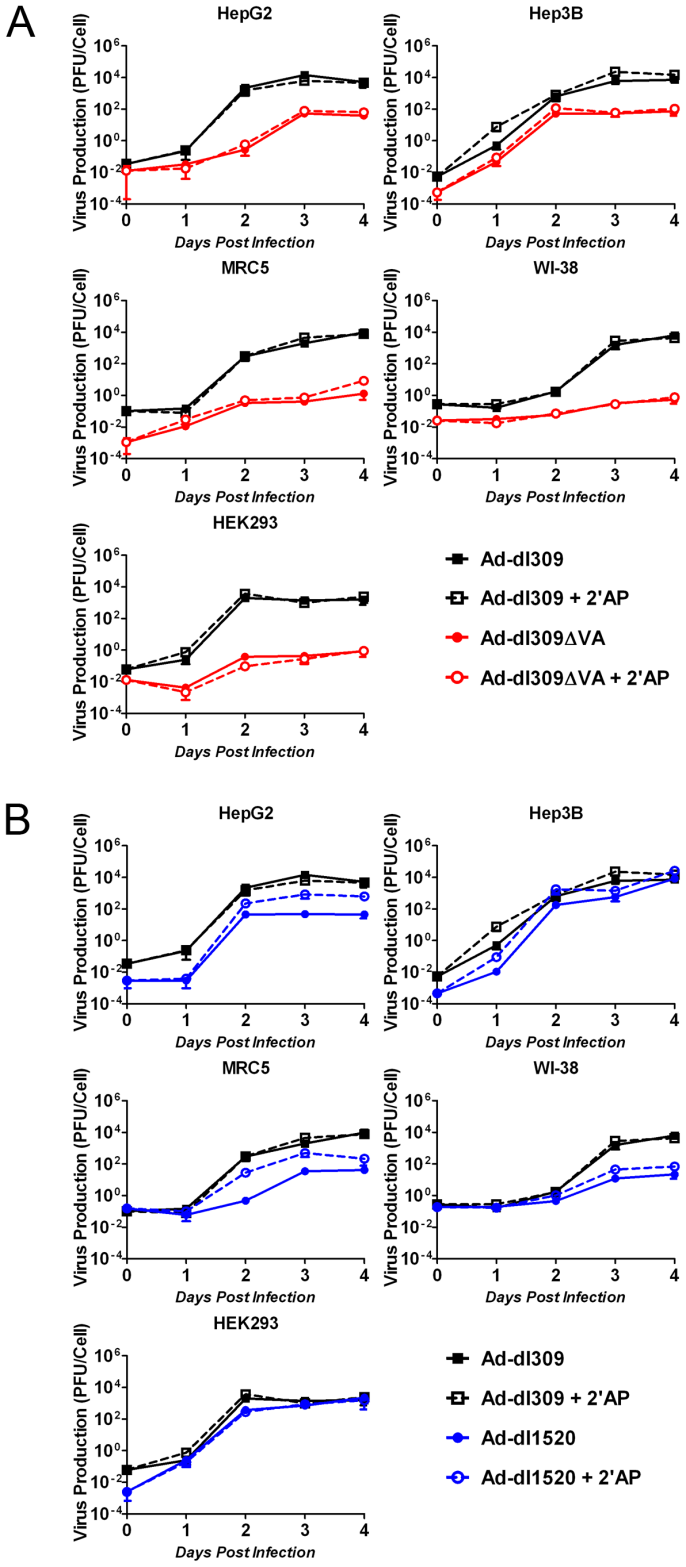
2′AP increases the replication of an adenovirus with an E1b-55K deletion but not with a VA-RNA deletion. Cells were infected in duplicate with (A) Ad-dl309**Δ**VA, (B) Ad-dl1520, or (A and B) Ad-dl309 at a multiplicity of infection (MOI) of 1 plaque forming unit per cell (PFU/cell) 1 hour prior to treatment with medium containing no drug or 2.5 mM 2′AP. Cells and media were harvested at 1 hr (day 0) as well as 1, 2, 3 and 4 days post-infection. Virus yields were determined using plaques assays on HEK293 cells. Error bars correspond to +/−SD.

**Figure 3:**
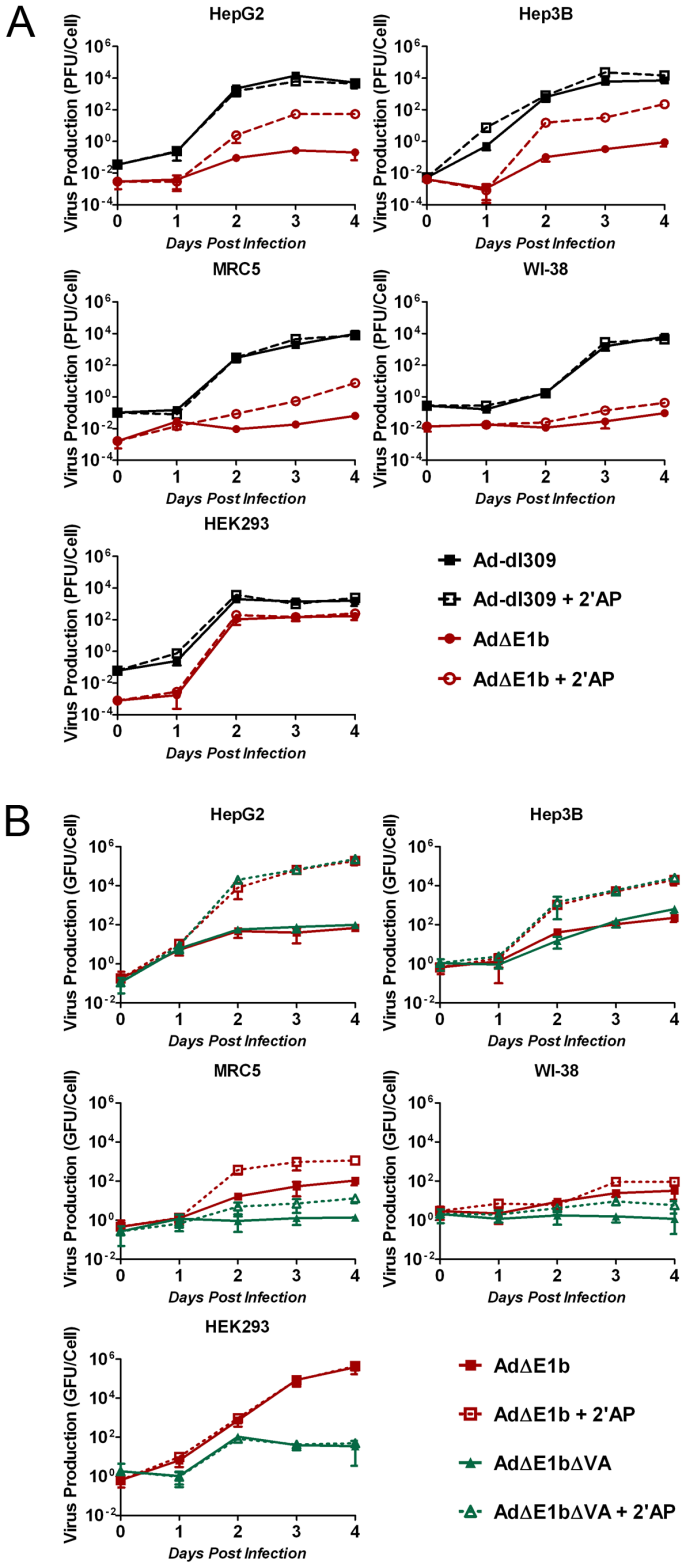
2′AP increases the replication of adenoviruses with an E1b deletion or with both E1b and VA-RNA deletions. (A) Cells were infected in duplicate with Ad-dl309 and Ad**Δ**E1b at an MOI of 1 PFU/cell 1 hour prior to treatment with medium containing no drug or 2.5 mM 2′AP. Cells and media were harvested at 1 hr (day 0) as well as 1, 2, 3 and 4 days post-infection. Virus yields were determined using plaques assays on HEK293 cells. (B) Cells were infected with Ad**Δ**E1b and Ad**Δ**E1b**Δ**VA at an MOI of 1 GFU/cell 1 hour prior to addition of medium containing no drug or 2.5 mM 2′AP. Cells and media were harvested at 1 hr (day 0) as well as 1, 2, 3 and 4 days post-infection. Virus yields were determined by titration in Hep3B cells. Error bars correspond to +/−SD.

In order to determine the effect of deletions of VA-RNA genes and/or E1b-55K on adenovirus growth, we generated a series of time-courses of virus production in a panel of hepatocellular carcinoma (HCC) and normal cell lines (for clarity, these data are presented in Figures 2 and 3). Ad-dl309 was used as a VA-RNA and E1b positive control (Figure 1), and therefore, its production time-course was plotted in both figures 2 and 3.

First, replication of viruses deleted in the VA RNA genes alone, or the E1b-55K coding sequence alone, were compared to Ad-dl309 (Figure 2A and 2B, respectively). Although the deletion of VA-RNA in Ad-dl309**Δ**VA was found to reduce adenovirus growth in both HCC and normal cells, the virus was more strongly attenuated in MRC5 and WI-38 normal cells when compared to HepG2 and Hep3B cells (4 and 2 orders of magnitude, respectively). Furthermore, Ad-dl309**Δ**VA was attenuated in HEK293 cells, which express proteins encoded by the E1 region of Ad5 [51]. Previously, 2′AP was shown to rescue the replication of a VA-RNA-deleted adenovirus [26], therefore, the panel of infected cells were also treated with 2.5 mM 2′AP. Surprisingly, 2′AP treatment did not have a significant effect on Ad-dl309**Δ**VA production in any of the tested cell lines.

Similar to the VA-RNA deletion (Figure 2A), the E1b-55K deletion in Ad-dl1520 also reduced virus growth in MRC5 and WI-38 normal cells when compared to Ad-dl309 (Figure 2B). However, the VA-RNA deletion had a stronger inhibitory effect on virus growth than the E1b-55K deletion in normal cells (4 and 2 orders of magnitude, respectively). Furthermore, while E1b-55K deletion had no effect on adenovirus production in Hep3B cells, the level of virus production was reduced in HepG2 cells. In addition, it should be noted that, although 2′AP treatment had no effect on Ad-dl309**Δ**VA production, the treatment appeared to slightly increase Ad-dl1520 production in HepG2, MRC5 and WI-38, suggesting that 2′AP could be partially compensating for lack of E1b-55K activities.

Taken together, these results suggest that Ad-dl309**Δ**VA may have higher selectivity for HCC cells than Ad-dl1520 does.

### Replication Properties of E1b-fully-deleted Adenoviruses in HCC and Normal Cells

Previous studies have shown that E1b-19K deletion may increase cancer-selectivity of E1b-55K deleted adenoviruses [24], [25]. Therefore, In order to increase the selectivity of E1b-55K-deleted adenoviruses for HCC cells compared to normal cells, we constructed an E1b-deleted adenovirus (Ad**Δ**E1b), which does not encode either E1b-19K or E1b-55K (Figure 1). Furthermore, due to a previous report showing that increasing E1a levels may increase the activity of E1b-55K-deleted-adenoviruses [49], we placed the E1a gene under the control of the strong immediate early murine cytomegalovirus (mCMV) promoter.

To examine the effect of these modifications on virus replication, we compared the growth properties of Ad**Δ**E1b to Ad-dl309 in the panel of HCC and normal cell lines (Figure 3A). We found that the deletions in Ad**Δ**E1b attenuated virus production in all the tested cell lines when compared to Ad-dl309. Similar to Ad-dl309**Δ**VA, Ad**Δ**E1b attenuation was stronger in MRC5 and WI-38 than in HepG2 and Hep3B. Interestingly, Ad**Δ**E1b production was also attenuated in the E1-positive HEK293 cells. This attenuation may be due to the larger deletion within the E3 region of Ad**Δ**E1b (encoding the adenovirus death protein gene) than that in both Ad-dl309 and Ad-dl1520 (Figure 1). Because we observed an increase in Ad-dl1520 production following 2′AP treatment (Figure 2B), we next tested whether 2′AP treatment could also increase Ad**Δ**E1b production. Interestingly, we found that 2′AP strongly increased Ad**Δ**E1b production in HepG2 and Hep3B cells, with a somewhat smaller effect in normal cells (Fig. 3A).

To further increase the selective production of E1b-deleted adenovirus in HCC cells relative to normal cells, we constructed an adenovirus deleted in both E1b and the VA-RNA genes (Ad**Δ**E1b**Δ**VA, Figure 1). Due to the low replication efficiency of Ad**Δ**E1b**Δ**VA in HEK293 cells as well as the inability of the virus to produce plaques in these cells, Ad**Δ**E1b**Δ**VA, and a stock of Ad**Δ**E1b for comparison purposes, were amplified in Hep3B cells. Furthermore, viral titers were determined using Hep3B cells and are expressed as “GFP-positive-cell forming units” per mL (GFU/mL). In order to determine whether the deletion of VA-RNA further attenuated virus production in normal cells, we generated a time-course of Ad**Δ**E1b and Ad**Δ**E1b**Δ**VA growth in the panel of cell lines (Figure 3B). Cells were infected at MOI of 1 GFU/cell and harvested at the indicated time points following infection. In HepG2 and Hep3B cells, the yields and growth rates of both viruses were similar, showing that the VA-RNAs are dispensable for the growth of Ad**Δ**E1b in these cells. In contrast, Ad**Δ**E1b**Δ**VA production was attenuated compared to Ad**Δ**E1b production in the normal cells, MRC5 and WI-38. As noted previously, Ad**Δ**E1b**Δ**VA production was highly attenuated in the E1-positive HEK293 cells. These results show that similar to Ad-dl309**Δ**VA, Ad**Δ**E1b**Δ**VA replication is selective for HCC cells relative to normal fibroblasts.

We next investigated whether 2′AP treatment of cells could compensate for the lack of both E1b and the VA RNAs during virus production (Figure 3B). The production of both Ad**Δ**E1b and Ad**Δ**E1b**Δ**VA were increased following 2′AP treatment in HepG2, Hep3B, MRC5 and WI-38 cell lines, but not E1b-positive HEK293 cells. Interestingly, we also found that the treatment of MRC5 and WI-38 with 2′AP did not increase production of Ad**Δ**E1b**Δ**VA to the same level as that of Ad**Δ**E1b, suggesting that 2′AP compensated for the activities lost by the E1b deletion rather than by the VA-RNA deletion in normal cells. Consistent with this hypothesis, 2′AP treatment did not have a significant effect on Ad**Δ**E1b**Δ**VA production in HEK293 cells that supply E1b activities in *trans*.

**Figure 4:**
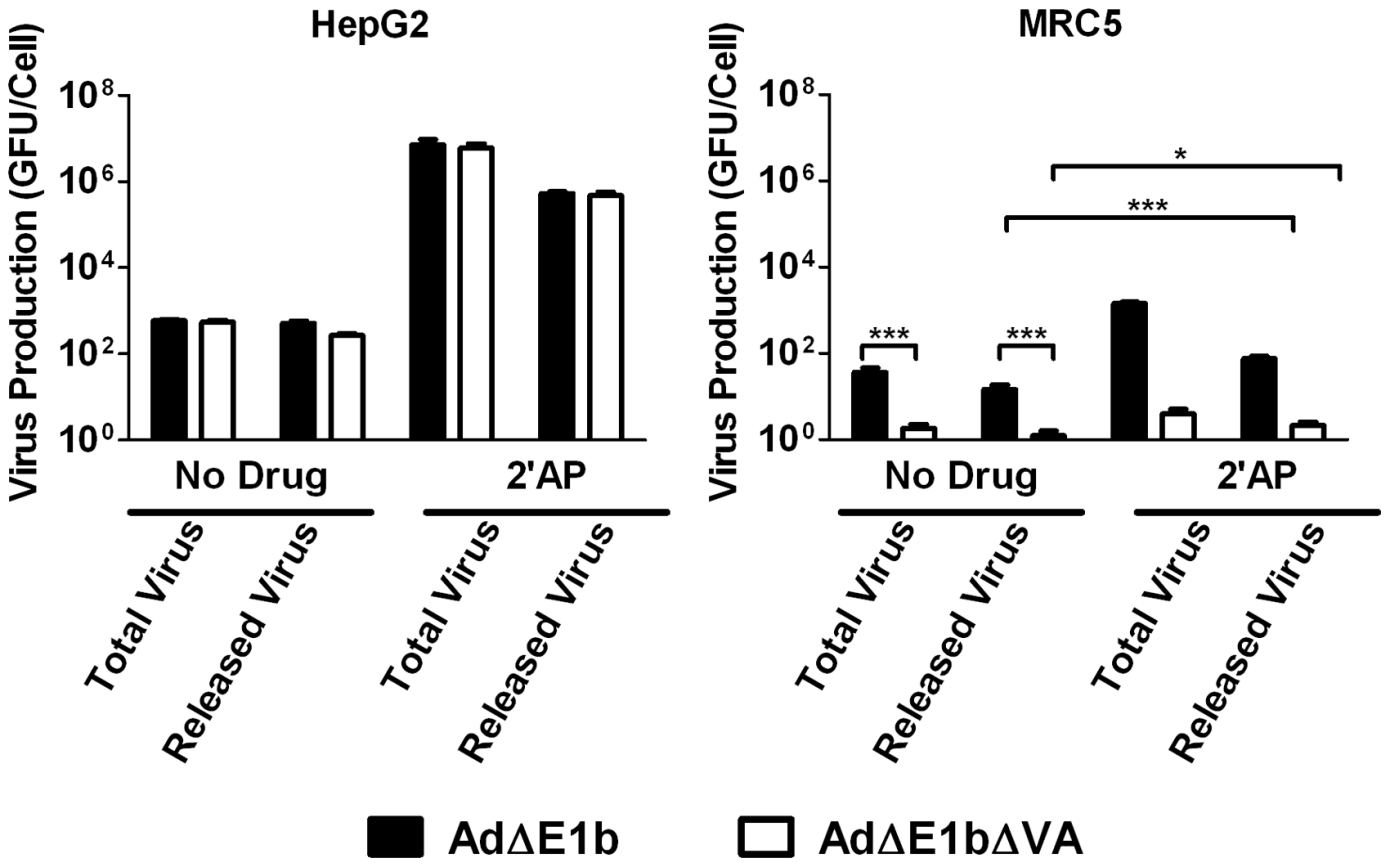
Treatment of HepG2 and MRC5 cells with 2.5 mM 2′AP inhibited AdΔE1b and AdΔE1bΔVA release. Cells were infected with either Ad**Δ**E1b or Ad**Δ**E1b**Δ**VA at MOI of 1 GFU/cell for 1 hour prior to treatment with medium containing no drug or 2.5 mM 2′AP. Four days following infection, infected cells combined with the media (total virus) or the media alone (released virus) were harvested. Virus yields were determined by titration in Hep3B cells. Error bars correspond to +/−SD of quadruplicates (*p<0.05, ***p<0.001, one-way ANOVA).

To examine the reported ability of 2′AP to reduce adenovirus release from infected cells [55], virus concentrations were measured from both the cells and media (total virus produced) as well as only the media (released virus) in HepG2 and MRC5 cells (Figure 4). In agreement with data shown in Figure 3B, we found little difference between the replication properties of Ad**Δ**E1b and Ad**Δ**E1b**Δ**VA in HepG2 cells at 4 days post-infection, whereas there was a significant difference between the two viruses in MRC5. Furthermore, 2′AP treatment of HepG2 increased production of both viruses by approximately 4 orders of magnitude. Production of both Ad**Δ**E1b and Ad**Δ**E1b**Δ**VA was also increased in MRC5 cells treated with 2′AP, however, this increase (10-fold) was much lower than in HepG2. Similar to a previous report [55], while most of the infectious virus particles were released when no drug was added, only approximately 10% of the virus was released from the HepG2 cells treated with 2′AP. Even though 90% of the virus was retained in cells treated with 2′AP, more virus was released following drug treatment than produced in untreated cells.

### 2′AP Treatment Increased Virally Encoded Protein Expression in HepG2 Cells Infected with the E1b-fully-deleted Viruses

Previous studies have shown that 2′AP can increase the translation of exogenous genes [56], [57], among its many other activities. Therefore, we examined E1a, fiber, and GFP expression in virus infected cells with and without 2′AP treatment. The objective was to assess whether an increase in E1a expression corresponded to expression of other virally encoded genes, as well as to virus production.

HepG2 and MRC5 cells were infected with Ad**Δ**E1b or Ad**Δ**E1b**Δ**VA at MOI of 100 virus particles (VP) per cell for 2 or 4 days, with or without treatment with 2′AP. The E1b- and VA-RNA-positive Ad-dl309 was used as a positive control, while Ad-dl309**Δ**VA and Ad-dl1520 were used as VA-RNA-deleted and E1b-55K-deleted controls, respectively. AdControl (non-replicating) as well as an uninfected sample were used as negative controls.

**Figure 5:**
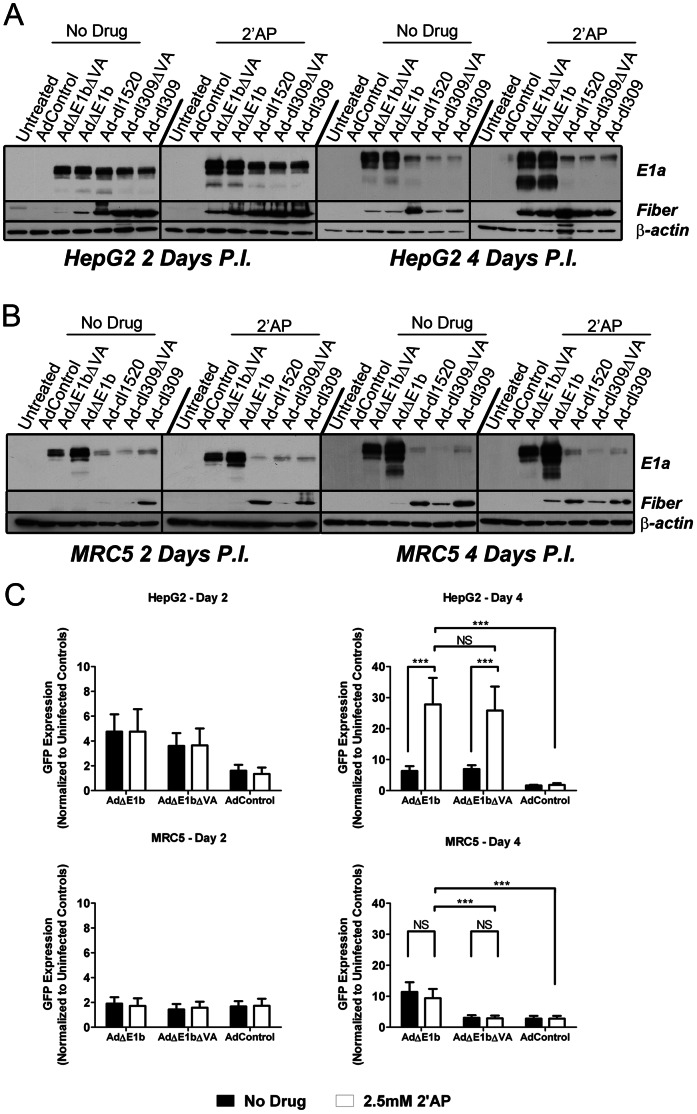
Treatment of HepG2 cells with 2′AP increased expression of virally encoded E1a, fiber and GFP. (A) HepG2 or (B) MRC5 cells were mock infected or infected with the indicated viruses at MOI of 100 VP/cell and then incubated for 2 days or 4 days, with or without 2.5 mM 2′AP treatment. Cells were washed and lysed and western blot analysis was performed on 10 µg protein per lane using antibodies against E1a, fiber and β-actin. (C) HepG2 and MRC5 cells were infected with Ad**Δ**E1b, Ad**Δ**E1b**Δ**VA or AdControl at MOI of 100 VP/cell and then incubated in the absence or presence of 2.5 mM 2′AP. Green fluorescence intensity was measured 2 and 4 days post-infection and normalized to uninfected controls. Error bars correspond to +/−SD of quadruplicates (NS – Not Significant; ***p<0.001, one-way ANOVA).

Two days after infection of untreated HepG2 cells with the E1b-fully-deleted viruses, E1a expression (under the control of the mCMV promoter) was similar to that in infections with Ad-dl309, Ad-dl309**Δ**VA and Ad-dl1520 viruses (under the control of the viral E1a promoter) (Figure 5A, left panel). By 4 days, much higher E1a expression levels were detected in HepG2 cells infected with the E1b-deleted viruses than in cells infected with the other replicating viruses (Figure 5A, right panel). This increase may be due to the loss of E1a autoregulation [58], as a consequence of replacement of the E1a promoter by the mCMV promoter, and/or due to activation of the mCMV promoter by E1a [59]–[61].

Treatment with 2′AP increased E1a levels in HepG2 cells infected with the E1b-deleted viruses at both 2 and 4 days post-infection. There could be a number of factors contributing to this observation. 2′AP may have increased E1a levels either directly through modifying the activity of the mCMV promoter controlling E1A expression in the E1b-deleted viruses, or indirectly through increasing virus replication thus increasing template copy number. In addition, 2′AP may have mediated increased translation [56], [57] of E1a.

In contrast to HepG2 cells, E1a levels in untreated MCR5 cells infected with the E1b-fully-deleted viruses were higher even at 2 days post-infection compared to cells infected with the other replicating viruses, suggesting that mCMV promoter is much more active than the E1a promoter in MRC5 cells (Figure 5B). Interestingly, 2′AP had little effect on mCMV promoter activity in MRC5 cells. Furthermore, E1a levels in MRC5 cells infected with Ad**Δ**E1b**Δ**VA were lower than in cells infected with Ad**Δ**E1b suggesting that the loss of VA-RNA genes reduced virus activity in these cells.

We also examined whether the expression of fiber, a late viral protein, corresponded to either E1a expression levels or to virus production in infected HepG2 cells. The absolute levels of E1a clearly did not correlate with fiber levels (Figure 5A). Nor did virus production correlate with absolute fiber levels, as all of the viruses except Ad-dl1520 had similar levels of fiber at day 4 in the absence of 2′AP, but replicated to very different levels at this time. However, if one considers relative changes induced by 2′AP, the increase in E1a expression mediated by 2′AP roughly correlated with an increase in fiber expression in infections of HepG2 cells with all of the replicating viruses (Figure 5A). With respect to virus production, however, the 2′AP-mediated increase in fiber levels in these cells only corresponded to increased growth of Ad-dl1520 and the E1b-fully-deleted viruses, and not Ad-dl309 or Ad-dl309**Δ**VA.

Similar to our observations with HepG2 cells, absolute E1a and fiber levels did not correlate in normal MRC5 cells (Figure 5B). It is notable that less fiber was produced in MRC5 cells infected with the E1b-fully-deleted viruses than with the other replicating viruses, paralleling virus production in these cells. Furthermore, increased fiber levels mediated by 2′AP treatment corresponded to increased virus production following treatment. Therefore, in contrast to HepG2 cells, fiber expression correlated closely with virus production in MRC5 cells.

To evaluate the activity of another virally encoded gene under these conditions, GFP expression (under the control of the hCMV promoter) was measured in HepG2 and MRC5 cells 2 and 4 days after infection with the E1b-fully-deleted viruses or the non-replicating AdControl (Figure 5C). As expected, GFP expression was low in AdControl infections of HepG2 cells at both time points, since this virus is non-replicating. Interestingly, we did not detect a significant difference in GFP expression between 2′AP-treated and untreated HepG2 cells infected with AdControl, suggesting that 2′AP did not activate the hCMV promoter. In contrast, GFP expression 4 days after infection of HepG2 cells with the E1b-deleted viruses was significantly increased by 2′AP treatment, similar to the results of E1a analysis. Taken together, these results suggest that the 2′AP-mediated increase in GFP expression may be due to increased viral DNA template rather than to increased hCMV promoter activity.

Similar to results of E1a analysis, 2′AP treatment of infected MRC5 cells did not have a significant effect on GFP expression. Also similar to E1a results, at 4 days post-infection, GFP expression was significantly lower in MRC5 cells infected with Ad**Δ**E1b**Δ**VA compared to Ad**Δ**E1b. Therefore, in contrast to results with HepG2 cells, we have found that 2′AP was unable to increase virus production or the expression of virally encoded E1a, GFP or fiber in Ad**Δ**E1b**Δ**VA-infected normal MRC5 cells to the same levels as in Ad**Δ**E1b-infected cells. This could have important implications for future therapies, as a drug similar to 2′AP might enhance activity of Ad**Δ**E1b**Δ**VA in HCC cells relative to normal cells.

### 2′AP Specifically Increased Virus-mediated HCC Cell Death

**Figure 6:**
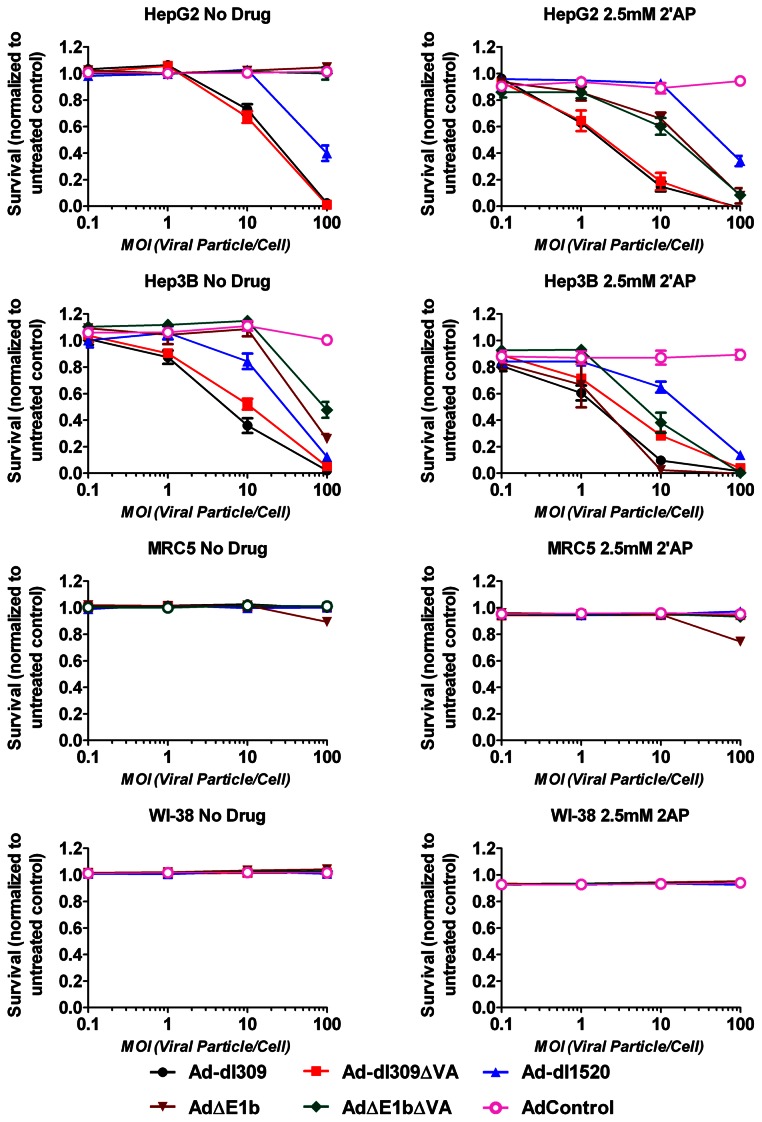
2′AP increased virus-mediated death of HepG2 and Hep3B HCC cells but not normal fibroblasts. HepG2 and Hep3B HCC cells as well as MRC5 and WI-38 normal cells were infected at the indicated MOIs and then incubated with or without 2.5 mM 2′AP. Six days post-infection, cell survival was measured by Alamar Blue fluorescence measurements and normalized to uninfected controls. Error bars correspond to +/−SD of quadruplicates.

The ability of 2′AP treatment to increase virus-mediated cell death was evaluated using the HCC cell lines, Hep3B and HepG2, and the normal cell lines, MRC5 and WI-38 (Figure 6). In the absence of 2′AP, HepG2 and Hep3B cells were found to be sensitive to Ad-dl309- and Ad-dl309**Δ**VA-mediated cell killing at MOI of 10 VP/cell. Furthermore, unlike HepG2 cells, Hep3B cells were also found to be sensitive to killing by the E1b-deleted viruses, although at a higher MOI. 2′AP treatment increased killing of both HepG2 and Hep3B cells by all of the viruses except Ad-dl1520. Interestingly, even in the presence of 2′AP, the normal cell lines were resistant to killing by all the replicating viruses, suggesting that the 2′AP increase in virus-mediated HCC cell death could be dependent on pathways specifically deregulated in HCC cells.

### 2′AP-mediated Virus Replication and Cell Death was Independent of p53 Levels or Activity

**Figure 7:**
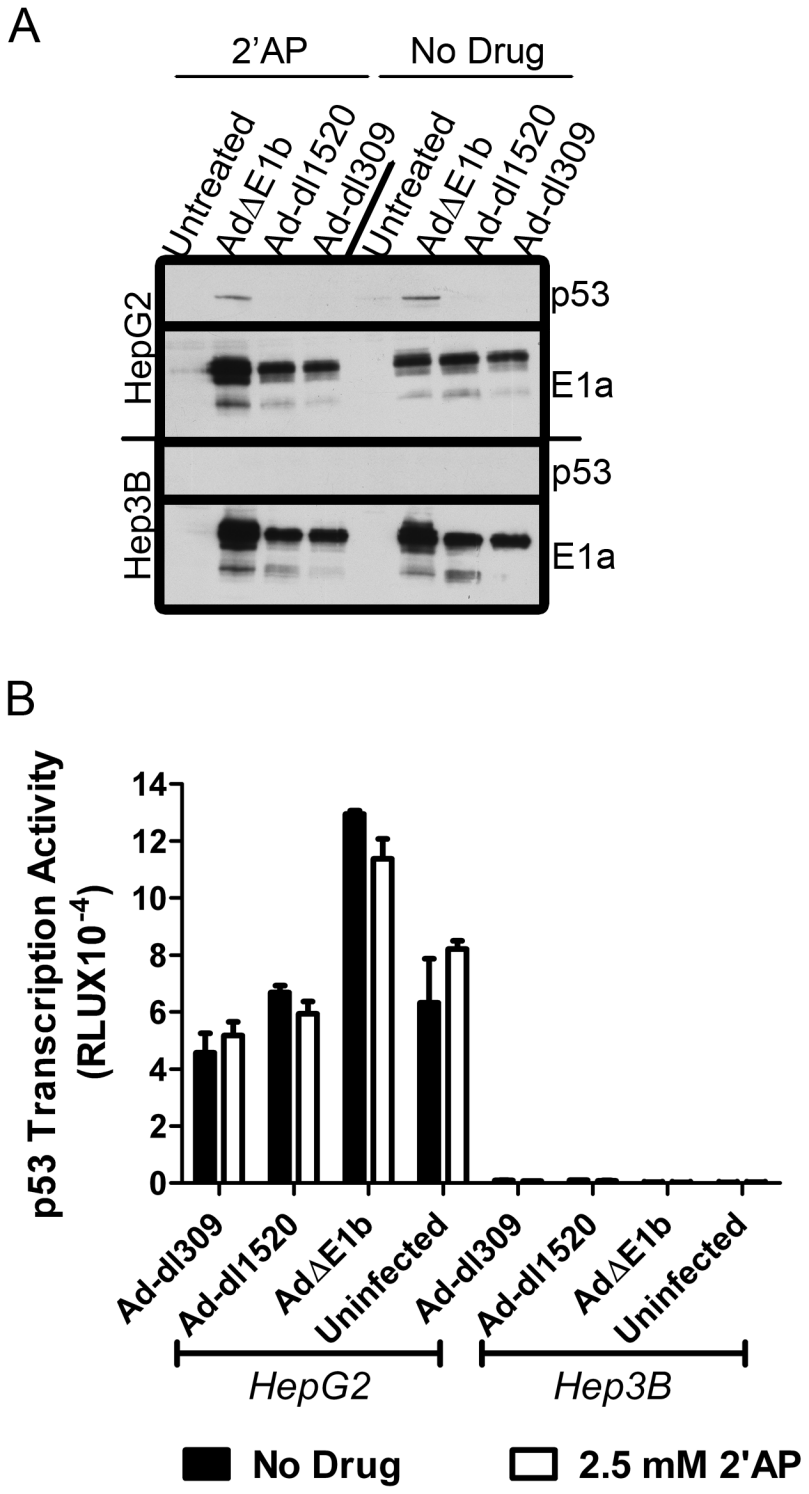
p53 expression and activity were not inhibited by 2′AP treatment of infected cells. (A) HepG2 and Hep3B cells were infected with Ad-dl309, Ad-dl1520 or Ad**Δ**E1b at MOI of 100 VP/cell and treated with no drug or 2.5 mM 2′AP for 2 days. p53 and E1a levels were detected by western blot analysis. (B) HepG2 and Hep3B cells were transfected with a p53-responsive firefly luciferase expression vector. The next day, transfected cells were mock infected or infected with Ad-dl309, Ad-dl1520 or Ad**Δ**E1b at MOI of 100 VP/cell and treated with no drug or 2.5 mM 2′AP. Luciferase expression was measured 2 days post-infection. Both HepG2 and Hep3B have high transfection efficiencies. Error bars correspond to +/−SD of triplicates.

One of the main functions of E1b-55K in an infected cell is to block p53 pathways, either by inhibiting p53 activity or decreasing its stability [2]–[4]. Here we examined the potential influence of p53 on replication of E1b-deleted virus in p53-positive HepG2 [62]. In HepG2 cells, p53 was only detectable in infections with Ad**Δ**E1b (Figure 7A). This is consistent with E1a-mediated stabilization of p53 [63]–[66]. However, we did not detect p53 in Ad-dl1520-infected HepG2 cells which had levels of E1a comparable to Ad**Δ**E1b infected cells at this time point. As Ad-dl1520 has previously been shown to induce or stabilize p53 levels [9], [67], [68], it is not immediately apparent why this virus did not enhance p53 protein levels in our system.

It has been reported [43] that 2′AP, like E1b-55K, inhibits p53 stabilization. Therefore, we examined whether the increase in E1b-deleted virus replication in response to 2′AP could be a result of alterations in p53 levels or activity. Contrary to our expectations, 2′AP treatment did not reduce p53 levels in E1b-deleted virus infected HepG2 cells (Figure 7A).

To further investigate the potential ability of 2′AP to alter p53 activity in this system, HepG2 cells were transfected with a p53-responsive reporter vector one day prior to infection with Ad-dl309, Ad-dl1520 or Ad**Δ**E1b (Figure 7B), then assayed for reporter gene expression 2 days later. Hep3B cells, used as a p53-negative control (Figure 7A and [69]), were unable to activate the p53-responsive reporter, demonstrating the specificity of this assay. In agreement with our analysis of p53 protein levels (Figure 7A), p53 transcriptional activity was higher in HepG2 cells infected with Ad**Δ**E1b than it was in infections with Ad-dl309 or Ad-dl1520. Furthermore, 2′AP treatment did not reduce the transcriptional activity of p53 in HepG2 cells infected with Ad**Δ**E1b. Taken together, these data suggest that the 2′AP-mediated increase in virus production was independent of p53 inhibition.

### 2′AP Specifically Rescued C454S/C456S Substitution of E1b-55K in HepG2

**Figure 8:**
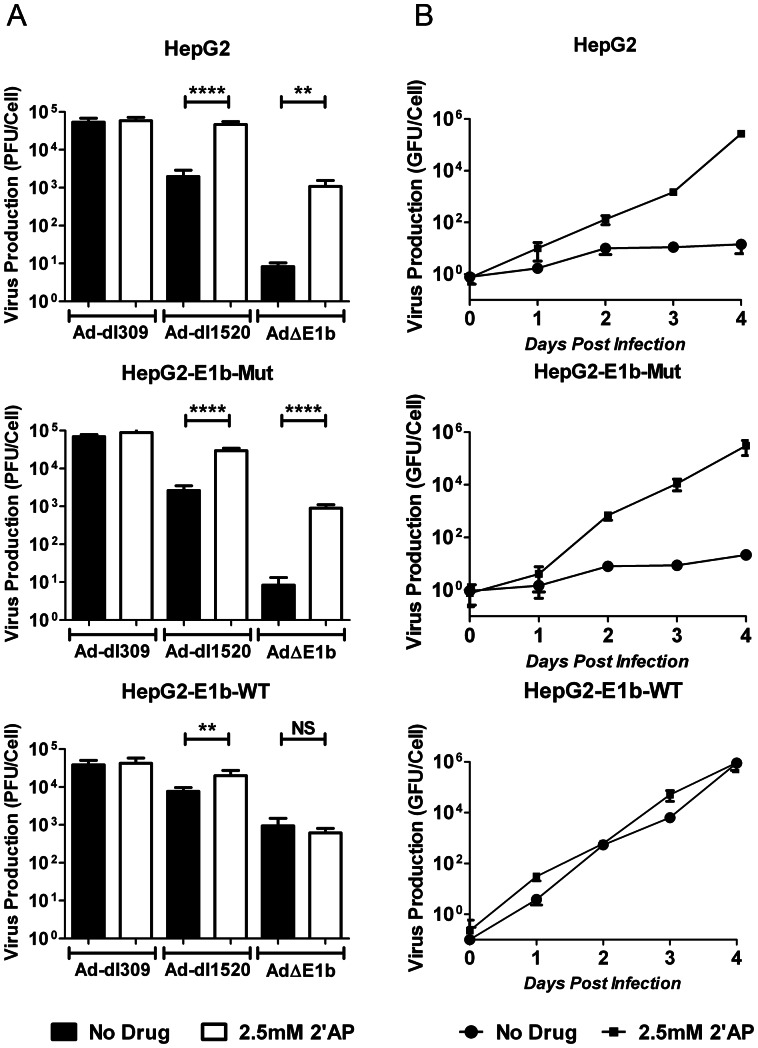
2′AP rescued the C454S/C456S E1b-55K mutation in HepG2 cells. (A) Parental HepG2, HepG2-E1b-WT and HepG2-E1b-Mut were infected with Ad-dl309, Ad-dl1520 or Ad**Δ**E1b at MOI of 1 PFU/cell for 1 hour prior to treatment with medium containing no drug or 2.5 mM 2′AP. Infected cells and media were harvested 4 days post-infection and virus yields were determine by plaque assays on HEK293 cells. (B) Cells were infected with Ad**Δ**E1b at MOI of 1 GFU/cell for 1 hour prior to treatment with medium containing no drug or 2.5 mM 2′AP. Lysates from infected cells were harvested at 1 hr (day 0) as well as 1, 2, 3 and 4 days post-infection. Virus yields were determined by titration in Hep3B cells as described in the materials and methods. Error bars correspond to +/−SD of quadruplicates (NS – Not Significant; ****p<0.0001, **p<0.01, one-way ANOVA).

Both 2′AP and E1b-55K have been shown to block the DNA damage response [13], [14], [44], [45]. Furthermore, previous studies have found that one of the E1b-55K domains responsible for this inhibition includes the residues C454 and C456 [17], [18]. Therefore, we tested whether inhibition of the DNA damage response could potentially be involved in E1b-deleted virus production in HepG2 cells treated with 2′AP. We stably transfected HepG2 cells with vectors encoding either wild-type E1b-55K or a mutated E1b-55K (C454S/C456S). The stably transfected HepG2 cells (HepG2-E1b-WT and HepG2-E1b-Mut) as well as the parental HepG2 cells were infected with Ad-dl309, Ad-dl1520 or Ad**Δ**E1b then cultured with or without 2′AP treatment for 4 days prior to virus production measurement (Figure 8A). While 2′AP had no effect on Ad-dl309 production in any of these cell lines, 2′AP strongly increased production of both Ad-dl1520 and Ad**Δ**E1b in parental HepG2 cells as well as HepG2-E1b-Mut cells. As expected, HepG2-E1b-WT at least partially complemented the E1b deletions in Ad-dl1520 and Ad**Δ**E1b allowing relatively high virus production, and 2′AP treatment did not further increase yields substantially. Interestingly, Ad**Δ**E1b production levels were much lower than Ad-dl1520 even in the complementing HepG2-E1b-WT cells, suggesting that other differences between Ad-dl1520 and Ad**Δ**E1b, such as E1b-19K and the E3 death protein encoded by Ad-dl1520, or mCMV-promoter-control of E1a in Ad**Δ**E1b, may also contributed to lower Ad**Δ**E1b production.

We also generated a time-course of Ad**Δ**E1b production with or without 2′AP in the stably transfected cells as well as the parental HepG2 cells (Figure 8B). Similar to the results in Figure 8A, we found that Ad**Δ**E1b production was dramatically increased by 2′AP treatment of the parental HepG2 as well as the HepG2-E1b-Mut cell line. Also, the level of Ad**Δ**E1b production in untreated HepG2-E1b-WT was similar to the production levels in 2′AP-treated parental HepG2 and HepG2-E1b-Mut, strongly suggesting that 2′AP can directly compensate for the activities mediated by C454 and C456 residues in E1b-55K.

### 2′AP Enhancement of Virus-mediated Cell Death was Independent of E1b-55K Expression

**Figure 9:**
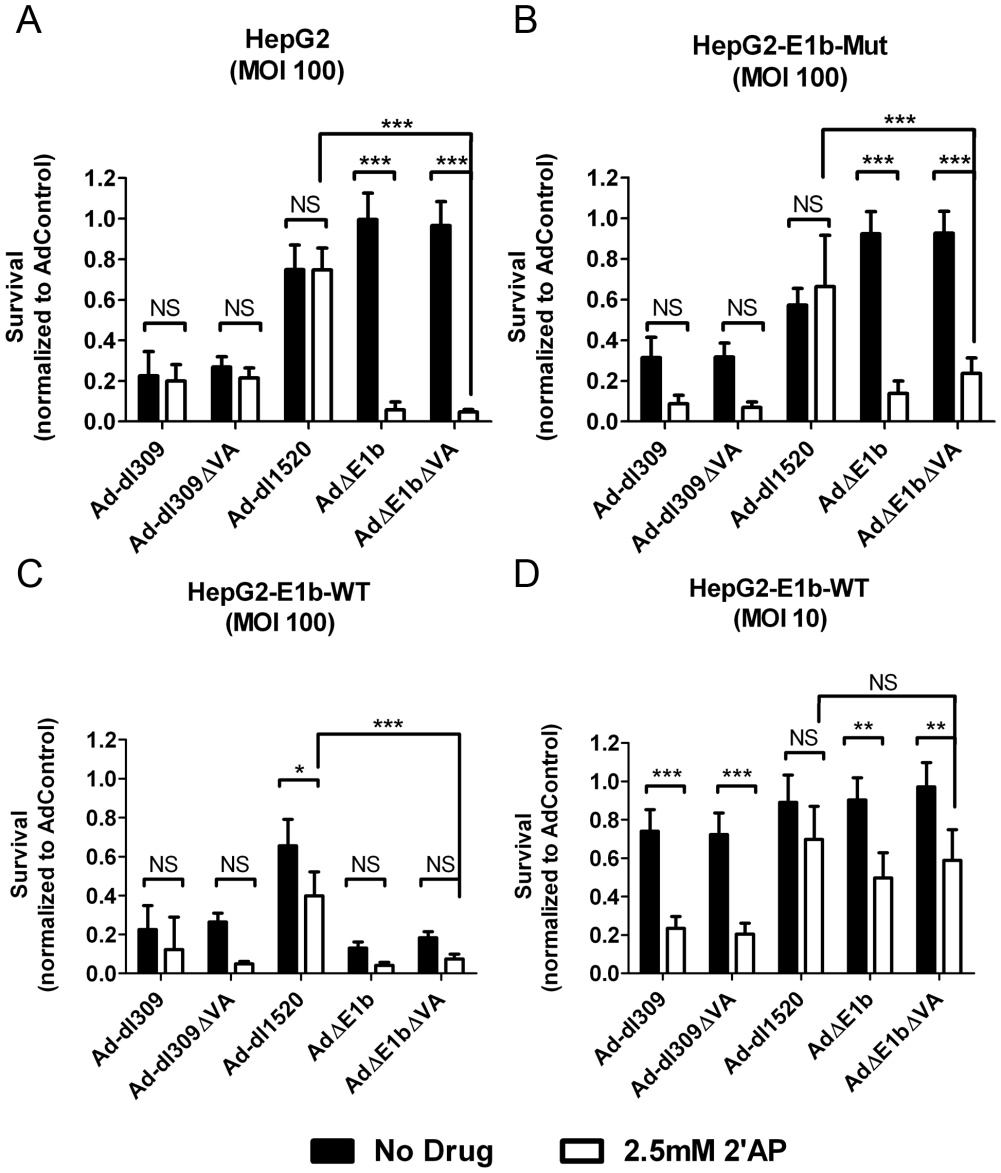
2′AP treatment increased both Ad-dl309 and AdΔE1b mediated HepG2 cell death. (A) parental HepG2, (B) HepG2-E1b-Mut and (C, D) HepG2-E1b-WT cells were infected with indicated viruses at MOI of 10 (D) or 100 (A, B, C) VP/cell and cultured in medium with or without 2.5 mM 2′AP. Survival was determined using an Alamar Blue assay 6 days post-infection. Fluorescence measurements were normalized to AdControl infected wells. Error bars correspond to +/−SD of quadruplicates (NS – Not Significant; ***p<0.001, **p<0.01, *p<0.05, one-way ANOVA).

Since 2′AP treatment and E1b-55K expression increased E1b-deleted virus production in a complementary manner, we investigated whether complementation would also be observed with virus-mediated cell death. HepG2-E1b-WT, HepG2-E1b-Mut and parental HepG2 cells were infected with the replicating viruses and incubated 6 days with or without 2.5 mM 2′AP prior to survival assessment (Figure 9). In the absence of 2′AP, efficient killing by virus at an MOI of 100 VP/cell (about 1 infectious unit/cell) required an intact E1b-55K protein either encoded by the virus (e.g., Ad-dl309 in Figure 9A) or provided in *trans* by the cells (e.g., Figure 9C). An activity of E1b-55K that is dependent on cysteine residues at 454 and/or 456 appeared to be critical for virus-mediated cell death, as the E1b-55K mutant protein did not complement the E1b-deleted viruses (Figure 9B). Furthermore, similar to its effect on virus production (Figure 6), 2′AP treatment could substitute for E1b-55K in killing by Ad**Δ**E1b and Ad**Δ**E1b**Δ**VA (Figure 9 A, B). In contrast to Ad**Δ**E1b, Ad-dl1520 did not induce greater killing of these cells when combined with 2′AP treatment, which may be due to lower E1a levels in 2′AP-treated cells infected with Ad-dl1520 compared to Ad**Δ**E1b.

At low virus concentrations (10 VP/cell; ∼0.1 infectious units per cell), a much smaller population of cells would be initially infected, resulting in a more stringent test for virus-mediated killing of HepG2-E1b-WT cells (Figure 9). Surprisingly, we found that 2′AP treatment increased killing by all the viruses except Ad-dl1520, suggesting that this drug induced deregulation of other cellular pathways in addition to those affected by the E1b-55K protein.

## Discussion

Oncolytic adenoviruses with a deletion of either E1b or VA-RNA genes have been extensively studied as potential cancer therapeutics. While these deletions increased the cancer-specificity of the replicating adenoviruses, these mutations often reduce their activity in both virus growth and cell lysis. Therefore, many studies have been performed to determine whether their activity could be increased by co-administration of chemotherapeutic drugs [8], [10], [26]–[28], [70]–[72]. 2′AP has recently been shown to increase the activity of an oncolytic herpes virus with deletions in several genes, including ICP34.5 [73]. Similar to E1b-55K and VA-RNAs, ICP34.5 was shown to target both the interferon response as well as the DNA damage response [74–77]. Therefore, we sought to determine whether the deletion of both E1b and VA-RNA genes could increase the selectivity of the Ad**Δ**E1b**Δ**VA for HCC cells, and whether 2′AP treatment could selectively compensate for the loss of E1b and/or VA-RNAs thus increasing the replicative and lytic activity of this virus.

To determine whether E1b-deleted virus replication was dependent on VA-RNA in HCC cells, we measured the growth rates of Ad**Δ**E1b and Ad**Δ**E1b**Δ**VA in both normal and HCC cells. We found that the VA-RNA deletion strongly attenuated Ad**Δ**E1b**Δ**VA production and lysis of normal cells, but not HCC cells. Our initial tests with 2′AP were designed to elucidate the mechanism of attenuation of the E1b-VA double-deleted virus. However, it was clear that 2′AP could not fully compensate for the combined deletion of VA-RNA and E1b-55K in normal cells although it greatly enhanced replication in HCC cell lines. The observation of differential enhancement in normal and HCC cells could have important implications for modulating tumor selectivity of the viruses studies here.

Our studies with Ad-dl1520 and Ad**Δ**E1b suggest that 2′AP complementation was more closely linked to the E1b deletion than to the VA-RNA deletion. Therefore, we investigated several reported activities of E1b-55K as potential candidates for the activity compensated by 2′AP in our infections. An important role of E1b-55K is blocking host protein synthesis [78],[79]. However, 2′AP is reported to inhibit the virus-mediated block in host protein synthesis [55], suggesting that 2′AP counteracts, rather than complements this E1b-55K activity. Furthermore, the 2′AP effect on protein synthesis resulted in reduced adenovirus release [55]. Consistent with this, we show here approximately a 10-fold reduction in virus release following 2′AP treatment of HepG2 and MRC5 cells.

Another role of E1b-55K is the promotion of late viral mRNA export [9],[80], which follows viral DNA replication. Consistent with this activity of E1b-55K, in MRC5 cells, increased fiber expression closely correlated to increased virus production. In contrast to MRC5 cells, 2′AP increased fiber expression in HepG2 cells infected with all the replicating viruses, and therefore, increased fiber expression did not correlate with increased virus production in these cells.

E1b-55K was reported to inhibit activation of the host DNA damage response mediated by newly synthesized viral genomes, thereby preventing genome concatemerization [14]. In order to determine whether adenovirus production in HepG2 cells might dependent on the inhibition of the DNA damage response, we measured the growth properties of Ad**Δ**E1b in HepG2 cells expressing either wild-type E1b-55K or mutant E1b-55K (C454S/C456S) that is unable to degrade DNA ligase IV and Mre11 [17],[18]. In contrast to wild-type E1b-55K, the mutant E1b-55K was unable to significantly increase Ad**Δ**E1b or Ad-dl1520 production. Furthermore, our data suggest that 2′AP compensated for the activity mediated by the C454 and C456 residues of E1b-55K. This activity was unlikely to involve p53 inhibition, since the E1b-55K mutant retains the ability to inhibit p53 [17],[18]. Consistent with this, we found that 2′AP treatment did not affect p53 levels or activity in Ad**Δ**E1b infected HepG2 cells, indicating that 2′AP compensation is not through p53 inhibition.

Although much of our data supports a mechanism for 2′AP enhancement of virus production and cell killing that recapitulates an E1b-55K activity, it is important to note that 2′AP had a stronger effect in infections with the E1b-fully-deleted viruses than with Ad-dl1520, suggesting that 2′AP activity may be more complex than simply compensating for E1b-55K.

In this report, we demonstrate that 2′AP could affect multiple pathways important for completing the adenovirus lifecycle. Furthermore, we show that 2′AP could not increase the replication of Ad**Δ**E1b**Δ**VA in normal MRC5 cells to the same level as Ad**Δ**E1b. Therefore, although E1b-55K and VA-RNAs may target similar pathways, the VA-RNAs also target pathways that are not complemented by either E1b-55K or 2′AP in normal cells. Further understanding of how 2′AP preferentially increases Ad**Δ**E1b**Δ**VA production as well as virus-mediated cancer cell death, could result in the design of new drugs that can activate attenuated adenoviruses only in cancer cells, thereby reducing toxicities associated with current oncolytic adenoviruses.
